# Maternal Age, Parity and Isolated Birth Defects: A Population-Based Case-Control Study in Shenzhen, China

**DOI:** 10.1371/journal.pone.0081369

**Published:** 2013-11-25

**Authors:** Ya Li Luo, Yu Li Cheng, Xiao Hui Gao, Shu Qin Tan, Jian Mei Li, Wei Wang, Qing Chen

**Affiliations:** 1 Department of Epidemiology, School of Public Health and Tropical Medicine, Southern Medical University, Guangzhou, China; 2 Department of Health Care, Baoan Maternal and Child Health Hospital, Shenzhen, China; 3 Department of Health Care, Shenzhen Maternal and Child Health Hospital, Shenzhen, China; 4 Faculty of Women's Health, Baoan Maternal and Child Health Hospital, Shenzhen, China; Tabriz University of Medical Sciences, Iran (Republic of Islamic)

## Abstract

**Background:**

The etiology of birth defects has been widely studied but is not yet fully clarified, previously published data had suggested that maternal age or parity maybe involved, but without consistent conclusions.

**Methods:**

A population-based, case-control study was nested in a cohort of perinatal infants born from 2010 to 2012 in Baoan District, Shenzhen. Four categories of isolated birth defects were defined as cases: congenital heart defects (CHD, n = 693), polydactyly (n = 352), cleft lip with or without palate (CL/P, n = 159) and equinovarus (n = 119). Controls were non-malformed infants (n = 11,307) randomly selected from the same area and period. Odds ratios (ORs) and the 95% confidence intervals (CIs) were computed by multivariable unconditional logistic regression analysis.

**Results:**

Young maternal age (<25 years old) was associated with a reduced risk of CHD (adjusted OR = 0.73, 95% CI 0.59–0.90), while with an elevated risk of polydactyly (adjusted OR = 1.42, 95% CI 1.09-1.84). Increased risk of CL/P-affected pregnancy was observed in mothers older than 35 years old (adjusted OR = 2.12, 95% CI 1.26–3.57). Compared to primipara, those having their second, and third or more delivery were less likely to have infants with equinovarus, with significant adjusted ORs of 0.59 (0.40–0.89) and 0.42 (0.19–0.93), respectively.

**Conclusion:**

Maternal age was significantly associated with CHD, polydactyly and CL/P relevant pregnancy. Mothers with higher parity might have lower risk of equinovarus occurrence in offsprings.

## Introduction

The etiology of non-chromosomal birth defects has been widely discussed but is not yet fully clarified, though numerous studies have confirmed that environmental and behaviors components are involved [1]–[6]. Apart from these influences, previous studies have implicated that maternal inherent characteristics, for instance, age [7]–[10] or parity [9]–[13] might be also responsible for certain categories of congenital defects. However, no consensus was reached, and results were usually inconsistent due to limited sample size and methodological difficulties.

Baoan district is accounted for the second largest area among the six functional districts in Shenzhen City, and accounted for the largest proportion (38.8%) of Shenzhen's total population (10.36 million) at the end of 2010 [14]. The average birth rate (15.88‰) and incidence rate of birth defects (14.14‰) were also reported higher than those of other districts [14].

As is generally accepted, population characteristics differ considerably across geographical location, therefore public health decision making should be based on the specific regional researches. However, etiologic studies regarding congenital defects in Chinese southern area have rarely been reported in recent years.

In the light of the above, we conducted a case-control study to investigate the relationship between maternal age, parity and selected isolated birth defects through a large cohort from Baoan district, Shenzhen, China.

## Methods

### Study Design

This study is a multi-center, population-based case-control study within a cohort of perinatal infants born in Baoan district from January 1^st^, 2010 to December 31^st^, 2012. Data utilized in this study were derived from Shenzhen Maternal and Child Health Management System (available on http://218.18.233.230:8888/), a continuously updated health care electronic database with an approximate 99.5% geographic coverage of the total area of Shenzhen. Anonymity and confidentiality were guaranteed, and written consents were given from the expectant parents, or their next of kin on the behalf of the newborn participants, for their information to be stored in the System at the time of the expectant mothers were awaiting delivery in the hospital. The study protocol was approved by the institutional review board of Baoan Maternal and Child Health Hospital.

### Case and Control Ascertainments

Perinatal infants (from 28 weeks' gestation to one week after birth) with congenital malformations were identified by Birth Defects Monitoring System (BDMS), a branch of Shenzhen Maternal and Child Health Management System.

In the present study, cases were defined as perinatal infants confirmed with isolated congenital heart defects (CHD), polydactyly, cleft lip with or without palate (CL/P) or equinovarus, which represented the top four phenotypes of birth defects in Baoan district. Those with multiple congenital defects, syndromic diseases or chromosomal aberrations were excluded as high genetic components were concerned [5].

The control pool was composed of all non-malformed perinatal infants born in the area from which the cases were recruited during the same period (2010–2012). Out of 203,154 records that met the primary criteria, 189,378 (93.2%) with complete information. The Power and Sample Size Calculations program (PS, version 3.0.43) was used to assess the minimum sample size of controls to guarantee a power of at least 79.6% (alpha 0.05, two-sided) to detect an OR of 2.0 or greater. Eventually a number of 11,305 records were randomly selected and constituted a share control group.

### Data Collection

Data on maternal and child records were prospectively entered into the Shenzhen Maternal and Child Health Management System. Once the cases and controls were ascertained, their basic characteristics were retrospectively reviewed and the selected covariates were extracted independently by two authors (Luo and Gao): general information of mothers (nationality, household registry, etc) and prenatal infants (gender, gestational age, birth weight, etc), the key exposure variables (maternal age and parity) as well as other covariates that were chosen to included in the analysis. Once the data extraction was completed, the outliers were rechecked by looking through the original medical records.

With rapid industrialization and urbanization, millions of Chinese peasants left their hometowns and headed to Shenzhen for job, making this city a famous modern industrial zone and worker-gathered area. For better management, Shenzhen government has classified the inhabitants' household registry into three types: 1) local resident for those who achieved permanent residence permit, 2) temporary population for those who lived in Shenzhen for more than one year without permanent residency and 3) migrant population for those who lived in Shenzhen for less than one year without permanent residency. The frequency of antenatal checkup was defined as the total numbers that had been taken through the whole gestation and treated as a categorical variable with four levels: 0, 1–5, 6–10 and >10. We also further grouped the checkup into “regular” and “irregular”. Regular checkup was defined as the first examination be taken from the 12^th^ weeks of gestation and more than five times be counted through the whole gestation.

In this study, maternal age was categorized into four groups: <25, 25–29, 30–34 and ≥35 years old. The second group was chosen as a reference because generally women of 25–29 years old has the lowest overall prevalence and represents the largest proportion. Parity was defined as the numbers of live birth a female has borne prior to the index pregnancy [15], and categorized into three levels: 0 (primipara, reference), 1 and ≥2.

### Statistical Analysis

For the enumeration data, independent sample *χ^2^* test or Fisher's exact test were performed to compare the status of case and control groups. Multivariable unconditional logistic regression analysis was used to compute the odds ratios (ORs) and the corresponding 95% confidence intervals (CIs) for the effects of maternal age and parity on the selected malformations. Two regression models were used: one crude model without any adjustments and another adjusted model that included adjustments for covariates that were deemed to be plausible confounders (covariates that were differentially distributed between cases and controls). Specially, liner regression analysis using a stepwise procedure was carried out to test co-linearity problems (eg. gestational age and birth weight) prior to multivariable logistic analysis. Variance inflation factor (VIF) ≥10 indicated strong co-linearity and then only one significant covariate was selected into the adjusted model. All statistical analyses were conducted by the SPSS, version 13.0 (Chicago, IL, USA). *P* value <0.05 was considered statistical significance.

## Results

### Cases and Controls Enrollments

A number of 693 CHD, 352 polydactyly, 159 CL/P and 119 equinovarus cases and 11,307 unaffected healthy controls were enrolled in the final analysis. The selected information of study subjects were summarized in **Table 1**.

**Table 1:** Basic characteristic of cases with isolated birth defects and unaffected controls.

Characteristics	CHD a	Polydactyly	CL/P ^b^	Equinovarus	Control
	(n = 693,%)	(n = 352,%)	(n = 159, %)	(n = 119, %)	(n = 11307, %)
Gender		_**_	_**_		
Male	396 (57.1)	228 (64.8)	102 (64.2)	59 (49.6)	6117 (54.1)
Female	297 (42.9)	124 (35.2)	56 (35.2)	60 (50.4)	5190 (45.9)
Gestational age (week)	_**_		_**_		
<32	46 (6.6)	3 (0.9)	11 (6.9)	2 (1.7)	72 (0.6)
32–37	185 (26.7)	52 (14.8)	38 (23.9)	21 (17.6)	1349 (11.9)
≥38	462 (66.7)	297 (84.4)	110 (69.2)	96 (80.7)	9886 (87.4)
Birth weight (gram)	_**_	_**_	_**_	*	
<2500	156 (22.5)	29 (8.2)	34 (21.4)	11 (9.2)	471 (4.2)
2500–3999	477 (68.8)	306 (86.9)	119 (74.8)	100 (84.0)	10269 (90.8)
≥4000	60 (8.7)	17 (4.8)	6 (3.8)	8 (6.7)	567 (5.0)
Delivery way	_**_		*		
Natural labor	385 (55.6)	223 (63.4)	121 (76.1)	70 (58.8)	7327 (64.8)
Cesarean	295 (42.6)	125 (35.56)	36 (22.6)	47 (39.5)	3891 (34.4)
Others	13 (1.9)	4 (1.1)	2 (1.3)	2 (1.7)	89 (0.8)
Plurality	_**_				
Single	668 (96.4)	345 (98.0)	158 (99.4)	116 (97.5)	11206 (99.1)
Twins	25 (3.6)	7 (2.0)	1 (0.6)	3 (2.5)	101 (0.9)
Outcome	_**_		_**_	_**_	
Live birth	625 (90.2)	349 (99.1)	133 (83.6)	115 (96.6)	11272 (99.7)
Still birth c	57 (8.2)	3 (0.9)	24 (15.1)	3 (2.5)	29 (0.3)
Neonatal death ^d^	11 (1.6)	0 (0)	2 (1.3)	1 (0.8)	6 (0)

a CHD: congenital heart defect, ^b^ CL/P: cleft lip with or without palate, one case for unknown gender.

c Fetal deaths of at least 28 weeks' gestation, ^d^ Died within one week after birth.

* *P* value <0.05, **P value <0.01 as compared with controls.

In many instances, there were statistically significant differences between case and control groups. A male predominance was noted among the polydactyly (64.8%) and CL/P (64.2%) cases as compared with controls (54.1%, *P*<0.05). Infants with CHD and CL/P in general had a lower birth weight and were more often prematurely born than those without malformation (*P*<0.01). The frequencies of still birth were also statistically higher in the CHD (8.2%), CL/P (15.1%) and equinovarus (2.5%) groups than those in the control group (0.3%). As the liner regression results had obviated the possibility of strong co-linearity (VIF = 1.439) between gestational age and birth weight, the above and other significantly incomparable covariates were considered to be biologically potential confounders and were entered into the adjusted model.

As listed in **Table 2**, more CL/P or equinovarus case-mothers fell into the “migrant population” group in comparison with control-mothers (76.7% vs. 61.7%, 74.8% vs. 61.7%, respectively). Significant lower frequency of antenatal checkup was also seen in CL/P-affected mothers when using 5 times as cut-off (85.5% vs. 68.6%). On the other hand, maternal pregnancy complications such as gestational diabetes mellitus or hypertension were sparse, thus most of the associations were detected as non-significant.

**Table 2:** Maternal factors of cases with isolated birth defects and unaffected controls.

Factors	CHD a	Polydactyly	CL/P b	Equinovarus	Control
	(n = 693, %)	(n = 352, %)	(n = 159, %)	(n = 119, %)	(n = 11307, %)
Nationality					
Han	634 (91.5)	327 (92.9)	147 (92.4)	111 (93.3)	10378 (91.8)
Other	59 (8.5)	25 (7.1)	12 (7.6)	8 (6.7)	927 (8.2)
Household registry	_**_		_**_	*	
Local resident	58 (8.4)	13 (3.7)	2 (1.3)	5 (4.2)	461 (4.1)
Temporary population	240 (34.6)	100 (28.4)	35 (22.0)	25 (21.0)	3867 (34.2)
Floating population	395 (57.0)	239 (67.9)	122 (76.7)	89 (74.8)	6979 (61.7)
Antenatal examination (num)			_**_		
0	52 (7.5)	27 (7.7)	19 (11.9)	4 (3.4)	742 (6.6)
1–5	397 (57.3)	221 (62.8)	117 (73.6)	75 (63.0)	7010 (62.0)
6–10	213 (30.7)	95 (27.0)	19 (11.9)	37 (31.1)	3119 (27.6)
>10	31 (4.5)	9 (2.6)	4 (2.5)	3 (2.5)	436 (3.9)
Pregnancy complication					
Gestational diabetes mellitus	12 (1.7) ^**^	0 (0)	0 (0)	0 (0)	37 (0.3)
Gestational hypertension	13 (1.9) *	3 (0.9)	0 (0)	3 (2.5)	96 (0.8)
Mild preeclampsia	4 (0.6)	0 (0)	2 (1.3)*	0 (0)	23 (0.2)
Severe preeclampsia	12 (1.7) ^**^	8 (2.3) ^**^	0 (0)	0 (0)	68 (0.6)

a CHD: congenital heart defect.

b CL/P: cleft lip with or without palate.

* *P* value <0.05, **P value <0.01 as compared with controls.

### Co-linearity test for maternal age and parity

As there exists a possibility that multi-parity is simply acting as a marker for maternal age, we also undertook liner regression analysis to test co-linearity problems, which were not demonstrated by the test results (VIF = 1.184), indicating that the impacts of parity were independent by maternal age.

### Maternal age and risk of selected isolated birth defects

**Table 3** **–** **4** showed the risk estimates of maternal age, parity in relation to the risk of delivering an infant with certain isolated birth defects. A protective effect on the occurrence of CHD was seen in younger mothers (adjusted OR = 0.73, 95% CI 0.59–0.90). On the contrary, observations of the polydactyly group indicated that mothers younger than 25 years old were more likely to have infants with polydactyly, albeit with modest association (adjusted OR = 1.42, 95% CI 1.09–1.84). A two-fold increased risk of having a CL/P-affected offspring was observed in mothers older than 35 years old (crude OR = 2.14, 95% CI 1.30–3.52) compared with those of 25–29 years old, and adjustments for covariates slightly decreased the risk estimates (adjusted OR = 2.12, 95% CI 1.26–3.57). There was no significant association between maternal age and equinovarus (for adjusted model: *P* = 0.11).

**Table 3:** Maternal age, parity and the risk of isolated CHD and Polydactyly.

Factors	CHD a			Polydactyly			Control
	(n = 693, %)	Crude OR	Adjusted OR	(n = 352, %)	Crude OR	Adjusted OR	(n = 11307, %)
		(95% CI)	(95% CI) b		(95% CI)	(95% CI) c	
Age (year)		_**_	_**_		*	_**_	
<25	159 (22.9)	0.73 (0.60–0.88)	0.73 (0.59–0.90)	123 (34.9)	1.43 (1.10–1.85)	1.42 (1.09–1.84)	4324 (38.2)
25–29	290 (41.8)	1.00 (Reference)	1.00 (Reference)	114 (32.4)	1.00 (Reference)	1.00 (Reference)	3550 (31.4)
30–34	164 (23.7)	1.03 (0.84–1.25)	1.03 (0.83–1.27)	88 (25.0)	1.40 (1.06–1.86)	1.42 (1.07–1.88)	2469 (21.8)
≥35	80 (11.5)	1.28 (0.99–1.66)	1.29 (0.98–1.70)	27 (7.7)	1.10 (0.72–1.69)	1.09 (0.71–1.66)	964 (8.5)
Parity (num)		*					
0	390 (56.3)	1.00 (Reference)	1.00 (Reference)	191 (54.3)	1.00 (Reference)	1.00 (Reference)	5837 (51.6)
1	236 (34.1)	0.80 (0.68–0.95)	0.84 (0.71–1.01)	130 (36.9)	0.90 (0.72–1.13)	0.88 (0.70–1.10)	4403 (38.9)
≥2	67 (9.7)	0.94 (0.72–1.23)	0.95 (0.71–1.28)	31 (8.8)	0.89 (0.60–1.31)	0.81 (0.54–1.20)	1067 (9.4)

a CHD: congenital heart defect.

b Adjusted for gestational age, birth weight, delivery way, plurality, outcome, household registry, gestational diabetes mellitus, gestational hypertension, severe preeclampsia, parity and age.

c Adjusted for gender, birth weight, severe preeclampsia and age.

* *P* value <0.05, **P value <0.01 as compared with reference group.

**Table 4:** Maternal age, parity and the risk of isolated CL/P and Equinovarus.

Factors	CL/P a					Equinovarus					Control			
	(n = 159, %)			Crude OR	Adjusted OR	(n = 119, %)		Crude OR		Adjusted OR	(n = 11307, %)			
				(95% CI)	(95% CI) b			(95% CI)		(95% CI) c				
Age (year)				_**_	*			*						
<25		61 (38.4)		1.61 (1.12–2.35)	1.29 (0.87–1.91)		47 (30.3)	1.73 (1.12–2.67)	1.41 (0.90–2.22)			4324 (38.2)		
25–29		50 (31.4)		1.00 (Reference)	1.00 (Reference)		36 (30.3)	1.00 (Reference)	1.00 (Reference)			3550 (31.4)		
30–34		25 (15.7)		0.91 (0.56–1.47)	0.89 (0.54–1.46)		22 (18.5)	1.11 (0.65–1.89)	1.25 (0.73–2.14)			2469 (21.8)		
≥35		23 (14.5)		2.14 (1.30–3.52)	2.12 (1.26–3.57)		14 (11.8)	1.81 (0.97–3.37)	2.13 (1.12–4.04)			964 (8.5)		
Parity (num)								*		_**_				
0		79 (49.7)	1.00 (Reference)		1.00 (Reference)		75 (63.0)	1.00 (Reference)	1.00 (Reference)			5837 (51.6)		
1		58 (36.5)	0.97 (0.69–1.37)		0.95 (0.66–1.37)		37 (31.1)	0.65 (0.44–0.97)	0.59 (0.40–0.89)			4403 (38.9)		
≥2		22 (13.8)	1.52 (0.95–2.46)		1.34 (0.81–2.23)		7 (5.9)	0.51 (0.24–1.11)	0.42 (0.19–0.93)			1067 (9.4)		

a CL/P: cleft lip with or without palate.

b Adjusted for gender, gestational age, birth weight, delivery way, outcome, household registry, antenatal examination, mild preeclampsia and age.

c Adjusted for birth weight, outcome, household registry, age and parity.

* *P* value <0.05, **P value <0.01 as compared with reference group.

### Maternal parity and risk of selected isolated birth defects

As outlined in **Table 3** **–** **4**, the adjusted analysis revealed a 41% and 58% decrease in the risk of delivering an infant with equinovarus among women giving birth to their second (OR = 0.59, 95% CI 0.40–0.97), and third or more (OR = 0.42, 95% CI 0.19–0.93) child respectively, as compared with primipara. Regarding CHD and polydactyly, reduced risks were also observed in higher parity groups, whereas without statistical significances. The crude model for CL/P pointed to a 52% increased risk for women with parity ≥2 (OR = 1.52, 95% CI 0.95–2.46), yet this risk estimate decreased and turned out to be non-statistical significant in the adjusted model, with an OR of 1.34 (95% CI 0.81–2.23).

## Discussion

In the current case-control study, we found that maternal age was significantly associated with CHD, polydactyly and CL/P relevant pregnancy, and those with higher parity were less likely to have equinovarus-affected offsprings.

The impacts of maternal age on birth defects have been long investigated, with inconsistent findings. An earlier study [16] conducted in the California population postulated that women 39 years or more of age were twice as likely as 25–29 years old to have a child with isolated CL/P, to which our study results were similar. Later Reefhuis and Honein [17] had carried out a large sample-size study using a cohort of 1,050,616 singleton infants born from 1968 to 2000 in Atlanta, and found that young maternal age (14–19 years old) represented a significant risk factor for cleft lip (OR = 1.88, 95% CI 1.30–2.73). However, a recent published meta-analysis [18] on oral clefts extracted data from 13 studied and suggested that, instead of early maternal age, mothers aged 40 years or over were 1.56 times more likely to have a newborn with non-syndromic CL/P (NSCL/P) compared with those aged between 20–29 years.

Meanwhile, Reefhuis and Honein [17] also observed that advanced maternal age (35–40 years old) was associated with all heart defects (OR  = 1.12, 95% CI 1.03–1.22). Similarly, Miller et al. [19] pointed an increased prevalence for several CHD phenotypes among women aging 35 years or older, with significant adjusted prevalence ratios ranged from 1.20 to 2.06. Yet our study did not replicate the above findings, on the contrary, we observed a reduced risk of CHD among younger mothers.

Unlike the widely explorations of CHD and CL/P, relatively little was reported about the impacts of maternal age on polydactyly and equinovarus. Our finding of a modest but significant association between young maternal age and a higher risk of polydactyly was in accordance with one previous study [17] that expectant mothers under 20 years old was documented to at higher risk of carrying a polydactyly offspring (OR  = 1.29, 95% CI 1.09–1.52). As for equinovarus, opposite conclusions had been drawn by two researches: Carey et al. [20] found that young maternal age (<20 years) was not associated with excess numbers of isolated talipes equinovarus cases while Parker et al. [21] observed 10% decreased risk in women younger than 23 years old in comparison with those of 23–34 years (Adjusted OR = 0.90, 95% CI 0.83–0.96). Nevertheless, our study did not replicate the above hypothesizes.

Similarly, it had not been verified whether maternal parity could contribute to the occurrence of certain birth defects. Recently Duong et al. [22] pointed that nulliparous mothers (those with no previous live birth) were more likely to have infants with tetralogy of Fallot and sepals cardiac defects compared with mothers with one prior live birth, with significant ORs of 1.34 and 1.20. In contrast, Messer et al. [23] had proposed that slightly larger proportion of NSCL/P cases were born to women with three or more prior births in Texas during 1999–2003. However, our data did not support the relation between parity and the above mentioned defects, and only obtained a significant reduced risk for equinovarus in multipara, which was opposite to the viewpoint that an excessive number of parturitions may lead to reproductive organ damage and dysfunction. Worth of note, the apparent protective effect of higher parity appeared to be of greater magnitude than that in previously report [21] which recruited 6,139 cases and 61,390 controls and showed significant adjusted ORs of 0.69 and 0.71 for birth order of second and third, respectively.

We deemed that nonstandard criteria for the age and parity grouping might be partially responsible for the inconsistent findings. Generally, the risk estimates for maternal age were calculated using 5-year categories with 25–29 years as the reference, while some studies introduced different classifications. For instance, Parker et al. [21] categorized the age into three groups with a wider range (<23, 23–34 and ≥35 years old). The definitions of parity were also various. Currently, the term of parity refers to the numbers of maternal parturition processes of live birth [15]. However, the common argument for parity is probably because of the definition of live birth, more exactly speaking, the “surviving fetus” (or fetus with vigor) has no unified criteria under different biomedical and technological levels. In the US, for example, those whose gestational age >20 weeks or birth weight >500 gram were considered as “substantially viable fetus”, while in the UK, parity is counted as the number of births beyond 24 weeks' gestation [24]. In China, this value was postponed to 28 weeks, which was also the lower limit value of perinatal period. As a consequence, there is, at the present time, no worldwide, uniform standard to define parity. In addition, selection bias from study arm, both cases and controls, could also be a possible explanation for discrepancies, since all the participants were recruited from different pools and therefore, population heterogeneities that were inherent in individual studies could not be ignored.

Our study had several notable methodological advantages, namely: 1) the sample size of the present study was comparatively large and substantial to decrease the rate of false-positive or false-negative of the results, the 95% CIs for risk estimates were relatively narrow and suggested sound statistical precision; 2) for the widely coverage and objectively data collection from the population-based register, both cases and controls could be considered as representative of the general population in Baoan district. Moreover, the selection and information bias was rendered to minimum; 3) multivariable adjusted analyses were conducted to reduce the errors introduced by confounders, just as the liner regression analysis for the co-linearity problems.

However, certain limitations were also needed to be acknowledged. Environmental conditions (such as occupational exposure of pollutants) or maternal certain personal behaviors (such as tobacco and alcohol consumption or nutritional deficiencies), as mentioned before, also had influence on the whole pregnancy outcome. Nevertheless, these information were not available for analysis, and it was difficult to derive such specific data because of the nature flaw of the Shenzhen Maternal and Child Health Management System, as the routine data collections were primarily used for administrative or governmental reports, most of the environmental and behaviors exposures were recorded as texts without uniform standard. Given the complexity of embryonic developments and the modest effect from single factor, it was possible that our findings were attributable to interactions among various components. Thus we stressed that the aforementioned results merited further comprehensive investigations.

In summary, our findings indicated that maternal age and parity might play an important role in the development of certain isolated birth defects. Given the prevalence of these defects in Baoan district, our findings might help other researchers to conduct more etiological studies and plan more comprehensive strategies to reduce the occurrence of birth defects in further generation.

## References

[1] Vaktskjold A, Talykova LV, Nieboer E (2011) Congenital anomalies in newborns to women employed in jobs with frequent exposure to organic solvents–a register-based prospective study. BMC Pregnancy Childbirth 11: 83. 22032401 10.1186/1471-2393-11-83 PMC3219734

[2] Desrosiers TA, Lawson CC, Meyer RE, Richardson DB, Daniels JL, et al (2012) Maternal occupational exposure to organic solvents during early pregnancy and risks of neural tube defects and orofacial clefts. Occup Environ Med 69: 493–499. 22447643 10.1136/oemed-2011-100245 PMC3719396

[3] Lorente C, Cordier S, Goujard J, Ayme S, Bianchi F, et al (2000) Tobacco and alcohol use during pregnancy and risk of oral clefts. Occupational Exposure and Congenital Malformation Working Group. Am J Public Health 90: 415–419. 10705862 10.2105/ajph.90.3.415 PMC1446183

[4] Honein MA, Rasmussen SA, Reefhuis J, Romitti PA, lammer EJ, et al (2007) Maternal smoking and environmental tobacco smoke exposure and the risk of orofacial clefts. Epidemiology 18: 226–233. 17202867 10.1097/01.ede.0000254430.61294.c0

[5] Anderka M, Mitchell AA, Louik C, Werler MM, Hernandez-Dias S, et al (2012) Medications used to treat nausea and vomiting of pregnancy and the risk of selected birth defects. Birth Defects Res A Clin Mol Teratol 94: 22–30. 22102545 10.1002/bdra.22865 PMC3299087

[6] Bailey LB, Berry RJ (2005) Folic acid supplementation and the occurrence of congenital heart defects, orofacial clefts, multiple births, and miscarriage. Am J Clin Nutr 81: 1213S–1217S. 15883454 10.1093/ajcn/81.5.1213

[7] Vieira AR, Orioli IM, Murray JC (2002) Maternal age and oral clefts: a reappraisal. Oral Surg Oral Med Oral Pathol Oral Radiol Endod 94: 530–535. 12424443 10.1067/moe.2002.128875

[8] Hollier LM, Leveno KJ, Kelly MA, MCIntire DD, Cunningham FG (2000) Maternal age and malformations in singleton births. Obstet Gynecol 96: 701–706. 11042304 10.1016/s0029-7844(00)01019-x

[9] Tay JS, Yip WC, Joseph R (1982) Parental age and birth order in Chinese children with congenital heart disease. J Med Genet 19: 441–443. 7154041 10.1136/jmg.19.6.441 PMC1048958

[10] Oddsberg J, Jia C, Nilsson E, Ye W, Lagergren J (2008) Influence of maternal parity, age, and ethnicity on risk of esophageal atresia in the infant in a population-based study. J Pediatr Surg 43: 1660–1665. 18779003 10.1016/j.jpedsurg.2007.11.021

[11] Zhan SY, Lian ZH, Zheng DZ, Gao L (1991) Effect of fathers' age and birth order on occurrence of congenital heart disease. J Epidemiol Commun H 45: 299–301. 10.1136/jech.45.4.299 PMC1059465 1795151

[12] Vieira AR, Orioli IM (2002) Birth order and oral clefts: a meta analysis. Teratology 66: 209–216. 12397628 10.1002/tera.10088

[13] Carmichael SL, Shaw GM, Laurent C, Olney RS, Lammer EJ, et al (2007) Maternal reproductive and demographic characteristics as risk factors for hypospadias. Paediatr Perinat Epidemiol 21: 210–218. 17439529 10.1111/j.1365-3016.2007.00809.x

[14] Health, Population and Family Planning Commission of Shenzhen Municipality (2010) Shenzhen Health Statistic Yearbook 2010. In: Luo LX, Chen GQ, editors. Basic demographic characteristics. Shenzhen: Shenzhen Press Group Publishing House. 37–38.

[15] Baird JT Jr, Quinlivan LG (1972) Parity and hypertension. Vital Health Stat 11: 1–28. 4536956

[16] Shaw GM, Croen LA, Curry CJ (1991) Isolated oral cleft malformations: associations with maternal and infant characteristics in a California population. Teratology 43: 225–228. 2014485 10.1002/tera.1420430306

[17] Reefhuis J, Honein MA (2004) Maternal age and non-chromosomal birth defects, Atlanta–1968–2000: teenager or thirty-something, who is at risk? Birth Defects Res A Clin Mol Teratol 70: 572–579. 15368555 10.1002/bdra.20065

[18] Herkrath AP, Herkrath FJ, Rebelo MA, Vettore MV (2012) Parental age as a risk factor for non-syndromic oral clefts: a meta-analysis. J Dent 40: 3–14. 22019990 10.1016/j.jdent.2011.10.002

[19] Miller A, Riehle-Colarusso T, Siffel C, Frias JL, Correa A (2011) Maternal age and prevalence of isolated congenital heart defects in an urban area of the United States. Am J Med Genet A 155A: 2137–2145. 21815253 10.1002/ajmg.a.34130

[20] Carey M, Mylvaganam A, Rouse I, Bower C (2005) Risk factors for isolated talipes equinovarus in Western Australia, 1980–1994. Paediatr Perinat Epidemiol 19: 238–245. 15860082 10.1111/j.1365-3016.2005.00648.x

[21] Parker SE, Mai CT, Strickland MJ, Olney RS, Rickard R, et al (2009) Multistate study of the epidemiology of clubfoot. Birth Defects Res A Clin Mol Teratol 85: 897–904. 19697433 10.1002/bdra.20625

[22] Duong HT, Hoyt AT, Carmichael SL, Gilboa SM, Canfield MA, et al (2012) Is maternal parity an independent risk factor for birth defects? Birth Defects Res A Clin Mol Teratol 94: 230–236. 22371332 10.1002/bdra.22889 PMC4476024

[23] Messer LC, Luben TJ, Mendola P, Carozza SE, Horel SA, et al (2010) Urban-rural residence and the occurrence of cleft lip and cleft palate in Texas, 1999–2003. Ann Epidemiol 20: 32–39. 20006274 10.1016/j.annepidem.2009.09.006

[24] Norwitz Errol R, Arulkumaran S, Symonds I, Fowlie A (2007) Oxford American Handbook of Obstetrics and Gynecology. New York: Oxford University Press. 760 p.

